# Outcome and upper extremity function within 72 hours after first occasion of stroke in an unselected population at a stroke unit. A part of the SALGOT study

**DOI:** 10.1186/1471-2377-12-162

**Published:** 2012-12-29

**Authors:** Hanna C Persson, Marina Parziali, Anna Danielsson, Katharina S Sunnerhagen

**Affiliations:** 1 Department of Clinical Neuroscience and Rehabilitation, Institute of Neuroscience and Physiology, Sahlgrenska Academy at the University of Gothenburg, Gothenburg, Sweden

**Keywords:** Stroke recovery, Upper extremity, Paresis, Outcomes, Process assessment

## Abstract

**Background:**

Reduced upper extremity function is one of the most common impairments after stroke and has previously been reported in approximately 70-80% of patients in the acute stage. Acute care for stroke has changes over the last years, with more people being admitted to a stroke unit as well as use of thrombolysis. The aim of the present study was to describe baseline characteristics, care pathway and discharge status in an unselected group of patients with first occasion of stroke who were at a stroke unit within 72 hours after stroke and also to investigate the frequency of impaired arm and hand function. A second aim was to explore factors associated with impaired upper extremity function and the impact of impairment on the patient’s outcome.

**Methods:**

Patients over 18 years of age with first ever stroke, living in a geographical catchment area, being at the stroke unit within 72 hours after onset, with no prior upper extremity impairment were included. Baseline characteristics, arm and hand function within 72 hours, stroke outcome and care pathway in the acute phase were described, by gathering information retrospectively from the patients’ charts. Ischemic strokes were categorized according to the Bamford classification and the Trial of Org 10172 in Acute Stroke Treatment criteria.

**Results:**

Of the 969 patients with first ever stroke who were screened, 642 patients fulfilled the inclusion criteria. According to the National Institutes of Health Stroke Scale (NIHSS), the patients had a mean score of 6.0, median 3.0, at arrival to the hospital. Ischemic stroke was most frequent in the anterior circulation (87.7%). Within 72 hours after stroke onset 48.0% of the patients had impaired arm and hand function and this was positively associated with higher age (p < 0.004), longer stay in the acute care (p < 0.001) and mortality in acute care (p < 0.001). Directly admitted to the stroke unit were 89.1% of the patients and 77.1% received hospital care on same day as stroke onset. Mean length of stay in the stroke unit was 9.9 days, 56.8% of the patients were discharged directly home from the stroke unit. Mortality within 72 hours after stroke onset was 5.0%.

**Conclusion:**

Impaired arm and hand function is present in 48% of the patients in a non selected population with first ever stroke, estimated within 72 hours after onset. This is less than previously reported. Impaired arm and hand function early after stroke is associated with higher age, longer stay in the acute care, and higher mortality within the acute hospital care.

## Background

The history of patients with stroke has changed [1]. In 2008, compared to 1995, it is more common that the patients are single, live at home needing assistance to a higher extent and have a better functional status prior the stroke [1]. Primary prevention is now more on the agenda, both on the individual level and on the population level. Acute stroke units and combined acute and rehabilitation units both seem to reduce the odds for dependency or death [2]. In Sweden, a stroke unit is defined as a designated unit at the hospital for acute stroke care with a team approach that includes rehabilitation staff, team meetings and discharge planning. A report [3] of stroke care in Sweden during 2007–2009 describes the patients’ care pathway; 66.4% of the patients are admitted directly to a stroke unit and 58% arrive at the hospital within three hours of stroke onset (63% within 4.5 hours). The acute care has also changed during the past decade [4], not only considering medical treatment, such as thrombolysis [5], but also regarding planning for discharge and rehabilitation, which starts even earlier.

Impaired motor function in the upper and lower extremities is reported to be as common as in 88% of patients [6] and influences the stroke unit care and the planning of the rehabilitation. Reduced upper extremity function is one of the most common impairments after stroke [7] and has previously been reported in approximately 70-80% of patients in the acute stage [7,8] assessed with the Barthel Index (BI), the Scandinavian Stroke Score [8] and a classification of impairments [7]. However, there is limited information on upper extremity function in the very first days after a stroke. When including patients in the ongoing Stroke Arm Longitudinal study at the University of Gothenburg, the SALGOT study [9], we noted that the number of patients with impaired arm and hand function seemed to be lower than previously reported.

The aim of the current study was to describe baseline characteristics, care pathway and discharge status in an unselected group of patients with first occasion of stroke who were at a stroke unit within 72 hours after stroke and also to investigate the frequency of impaired arm and hand function. A second aim was to explore factors associated with impaired upper extremity function and the impact of impairment on the patient’s outcome.

## Methods

All patients with first ever clinical stroke admitted to one of three comprehensive stroke units at Sahlgrenska University Hospital in Gothenburg, Sweden, during a period of 17.5 months, between February 4, 2009 and December 2, 2010, with two breaks (in total 145 days) for administrative reasons, were included. This stroke unit receives all candidates for thrombolysis and interventional radiology. Data were retrospectively gathered from the hospital data system as well as from the patient’s medical charts. The inclusion criteria for the study were: 1) first ever clinical stroke, defined according to WHO criteria [10] by either imaging or clinical assessment, 2) being at the stroke unit within 72 hours after stroke onset, 3) over 18 years old, 4) living in the geographical catchment area (within 35 km from the hospital), 5) no prior upper extremity impairment. From the hospital records eligible patients were identified according to the WHO’s International Classification of Diseases 10; diagnosis codes I61 and I63. The patient charts were then screened and first clinical stroke diagnosis was confirmed. Also from the patient's charts, documented by physicians or physiotherapists at the stroke unit, prior impaired upper extremity function limiting functional use of the affected arm and hand, such as orthopaedic, rheumatologic or neurological impairments was reviewed. Ischemic strokes were classified for subtype using the Bamford classification [11] and the cause of ischemic lesion by using a classification by the Trial of Org 10172 in Acute Stroke Treatment, (TOAST) [12]. The upper extremity function was evaluated and defined as impaired or not impaired based on the following information from the patient’s chart: 1) a documented assessment performed with the Modified Motor Assessment Scale (M-MAS UAS −95) [13] within 72 hours after stroke onset by one of the physiotherapists working at the stroke unit 2) evaluation of other documented standardized assessments of upper extremity function performed by the physiotherapists, occupational therapists or physicians at the stroke unit, within 72 hours after stroke onset. These data from the charts, were assessed independently by the first (HCP) and second (MP) author. If there were differences between the two, a new assessment was performed by HCP. If HCP:s opinion differed from the first assessment then a third reading of the chart was made to reach a decision.

From the M-MAS UAS-95 [13] a sub score including an arm function test, a grip test and a pinch test where both the affected and non affected arms are tested was used. Impaired function in the affected arm was defined as a reduced score in at least one of three items. To validate this classification of upper extremity function, a comparison was made with the arm score of the National Institutes of Health Stroke Scale (NIHSS) [14] performed at arrival to the hospital.

From the patient’s charts, the discharge status from the stroke unit was evaluated using the modified Rankin Scale (mRS) [15]. Data on living condition before stroke, medical treatment, secondary complications and care pathway for every patient during the stroke unit stay were taken from the patient’s charts and from the Swedish Stroke Register [16]. Since data were gathered for clinical purposes and quality control, according to Swedish law on personal particulars data from 1998 (Personuppgiftslagen, Swedish law No. SFS 1998:204), no informed consent was needed for this specific study. The study was approved by the Regional Ethics Committee, Gothenburg.

### Statistics

Non-parametric statistics were used since we did not assume a normal distribution of data. Cross tabulation and the Mann–Whitney U-test were applied to assess differences between independent groups and the Spearman rank-order correlation to investigate correlations. Descriptive statistics were used to show the number of patients who had impaired arm and hand function. A 2-tailed significance level of 0.05 was used for all tests.

## Results

**Figure 1:**
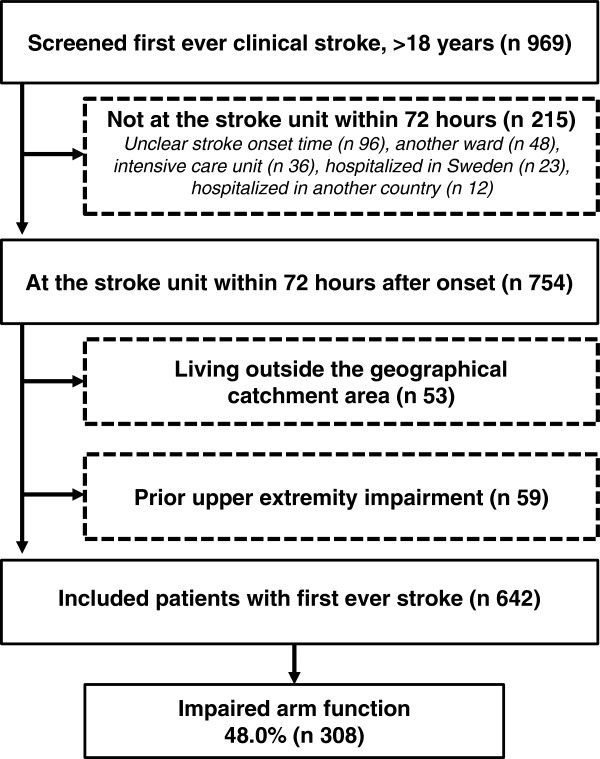
A schematic description of the screening and inclusion process and the frequency of impaired arm and hand function in patients available at the stroke unit within 72 hours after stroke onset.

During the study period 969 patients with first ever clinical stroke (55.9% men) 18 years and older were admitted to the stroke unit and screened for inclusion to the study. The results of this process are described in Figure 1. The main characteristics of the included 642 patients (average 73 years) are presented in Table 1. Lacunar anterior circulation infarct (LACI) was the most frequent type of ischemic stroke (44.0%). The results of the Bamford classification and the TOAST classification are presented in Table 1 along with information on acute interventions given. The length of hospital stay and care pathway are presented in Table 2, and most patients (77.1%), were admitted to the hospital on the same day as the stroke onset. The majority (89.1%) of the patients (n 642) were directly admitted to the stroke unit on arrival to the hospital and the mean length of stay in the stroke unit was 9.9 days (SD 8.8, range 1–61). Mortality within 72 hours after stroke onset was 5.0% (32 patients) and a further 4.2% (27 patients) died within ten days. At discharge from the stroke unit, 63.9% of the 571 patients who survived went directly to their home with or without homecare and the other patients required further hospital based rehabilitation.

**Table 1:** Study population description (n = 642)

**Characteristics**		**n**	**%**
**Male/Female**		351/291	54.7/45.3
**Age, years**	Mean 72.7, SD 14.2		
**Pre stroke**	*At home, without/with homecare*	526/85	81.9/13.3
**living condition**	*At nursing home/other*	24/7	3.7/1.1
	*Alone/together with another person*	295/347	46.0/54.0
**Pre stoke**	*Mobility independent*	589	91.7
**functional ability**	*Dependent outdoors*	46	7.2
	*Dependent in- and outdoors*	7	1.1
	*Bladder bowel independent/dependent*	625/17	97.4/2.6
	*Dressing independent/dependent*	623/19	97.0/3.0
**Hemisphere of stroke**	Right/left/bilateral/unclear	264/293/30/55	41.1/45.6/4.7/8.6
**Type of stroke**	*Ischemic infarct*	575	89.6
	*Intracerebral haemorrhage*	67	10.4
Type of ischemic stroke, Bamford*	*Lacunar anterior cerebral inf, LACI*	253	44.0
	*Partial anterior cerebral inf, PACI*	204	35.5
	*Posterior circulation inf, POCI*	71	12.3
	*Total anterior cerebral inf, TACI*	47	8.2
		
TOAST classification, ischemic stroke*	*Small artery occlusion, SAO*	223	38.8
	*Cardio-aortic embolism, CAE*	184	32.0
	*Large-artery atherosclerosis, LAA*	101	17.6
	*Other causes, OC*	26	4.5
	*Undermined causes, UND*	14	2.4
	*Unclear, UNCL*	27	4.7
**Treatment**	*Thrombolysis*	57	8.9
	*Thrombectomy*	22	3.4
	*Hemicraniectomy*	1	0.2
NIHSS, (0–42 p)*	Mean 6.0, SD 6.8	345	
	Median 3.0, Range 0-33		
Consciousness*	*Fully conscious*	540	84.1
	*Mildly confused or drowsy*	70	10.9
	*Unconscious*	32	5.0
**Modified Ranking Scale, (0–6) at discharge from stroke unit**	0	3	0.5
	1	51	7.9
	2	170	26.5
	3	185	28.8
	4	151	23.5
	5	12	1.9
	6	70	10.9

* At arrival to hospital.

*Inf*, infarct; *NIHSS*, National Institutes of Health Stroke Scale; *SD*, standard deviation; *TOAST*, the Trial of Org 10172 in Acute Stroke Treatment.

**Table 2:** Hospitalization time and care pathway (n = 642)

**Characteristics**	**n**	**%**
**Hospital care, first admitted to**		
*Stroke unit*	572	89.1
*Acute ordinary ward*	52	8.1
*Intensive care unit*	18	2.8
**Discharge to**		
*Home*	365	56.9
*Geriatric or rehab ward*	136	21.2
*Nursing home*	53	8.3
*Other acute ward*	17	2.6
*Ad mortem*	*71*	*11.1*
**Complication during Stroke Unit care**		
*Pneumonia*	32	5.0
*Fracture*	6	0.9
*DVT*	1	0.2

*DVT*; deep vein thrombosis, *Rehab*; rehabilitation.

As presented in Figure 1, 48.0% had impaired arm and hand function within the 72 hours after stroke onset. The majority, 80.4%, of these 642 were assessed with M-MAS UAS-95. The comparison between the evaluations of arm and hand function measured within 72 hours after onset (assessed at median 1.5 days post admittance to hospital) and upper extremity function at arrival to hospital (NIHSS) showed similar results in 77.6%.

Age was significantly associated with impaired arm and hand function (p < 0.004), i.e. older patients had more often impaired arm and hand function. In this study, no significant difference was noted regarding upper extremity function between those patients who received thrombolysis (8.8%) compared to others. Nor did time of arrival to hospital significantly associate with the arm and hand function. No significant difference between men and women regarding impaired upper extremity function was seen. However, impaired arm and hand function was significantly more frequent in patients with total anterior cerebral infarct (TACI) as compared to lacunar anterior cerebral infarct (LACI) (p < 0.001) and also among patients with TACI as compared to partial anterior cerebral infarct (PACI) (p < 0.028).

The association of the impaired upper extremity function on the patient’s care pathway was explored. Having an impaired upper extremity function within 72 hours after onset was significantly associated with higher mortality during the stroke unit stay (p < 0.001). Of the patients who survived (n 571) the stroke unit stay, patients with impaired arm and hand function had a longer length of stay at the stroke unit (p < 0.001), in mean 13.5 days compared to 7.8 days for patients with no reduced function, and were also more seldom discharged directly to the home (p < 0.001). Of those patients with impaired arm and hand function who survived, only 39.0% (n 96) were discharged directly home compared to 82.8% (n 269) with no reduced upper extremity function.

## Discussion

This article presents 642 patients with first ever clinical stroke, their upper extremity function within 72 hours after stroke onset, their baseline characteristics and their outcome and care pathway before discharge from a stroke unit. As reported in this study, 48.0% of the patients had impaired arm and hand function due to stroke as assessed within 72 hours after onset. This is less than the previously reported 70-80% in the acute stage [7,8]. Another purpose of this study was to explore the association between impaired arm and hand function and outcome in the acute stage. The mortality was significantly higher (p < 0.001) in patients with impaired upper extremity, and stroke survivors with impaired arm and hand function needed stroke unit care significantly longer and were also more seldom discharged directly to their home as compared to patients with unaffected upper extremity function. Compared to patients with LACI and PACI, patients with TACI more commonly had impaired arm and hand function which is also confirmed by Lawrence et al. [7].

Baselines characteristics in this non selected group of patients with first ever stroke were also explored. The average age at stroke accident was 73, compared to the Swedish national average of 76 years. Patients in the present study also had better function prior to the stroke compared to other studies in Sweden [1,17], which also include patients with recurrent stroke, which might explain the difference. The in-hospital care duration in the stroke unit is slightly shorter in the present study compared to the Swedish national average (10.3 days) for stroke unit stay [3].

The study includes all patients, from different socioeconomic groups, who are offered care on the same conditions, additional to ensure a well defined group; patients not living in the geographical catchment area were excluded. This improves the results for the present study and makes them more generally applicable to other stroke populations. The study covers one stroke unit, where patients had to be admitted within 72 hours of the stroke event, which mainly excludes patients at intensive care units or neurosurgery wards.

The fact that less than 50% of patients with acute stroke had an impaired arm and hand function was to some extent surprising. Reasons may be that the stroke care has changed in recent years; all patients with suspected stroke should be examined in hospital (including minor stroke) and both the rehabilitation and the medical treatment have improved. Also the primary prevention has changed, with better treatment for hypertension and dyslipidemia and fewer persons are smoking compared to the early 1990s. Earlier studies [6,8] are based on data gathered more than 15 years ago, include assessment of both upper and lower extremities [6], or are based on a later time for evaluation of motor function [6-8] and may not be representative of today’s patients. Furthermore, the current study includes only first ever stroke, which is not the case in previously mentioned studies [6,8], (personal communication with Skyhoj Olsen [8]). A limitation of the present study is the retrospective data collection, which may leads to incomplete data sets. For example, to evaluate arm and hand function, a sub score from M MAS-UAS −95 [13] was used, which is commonly used in the clinic to assess function. In this sample, 80% of the patients M MAS-UAS −95 results were documented in the charts. The remaining 20% of the patients’ upper extremity function was evaluated by reading the patients’ charts in a standardized way. This completion of the data set makes it possible to assess upper extremity function in an unselected population and give a representative group of patients from the specific geographical catchment area.

The agreement between the assessments of upper extremity function measured within 72 hours (M MAS-UAS-95) and at arrival to the hospital (NIHSS) was 78%. The 22% that were assessed as different could be explained by the fact that the assessments had taken place at different time points and the patients function could have changed between the assessments. However, this evaluation of upper extremity function seems more accurate than prior studies in which BI was used to estimate upper extremity function [7,8]. The M MAS-UAS-95 assesses at a body functional level whereas BI is primarily an activity measurement where compensation is possible and specific upper extremity function is not reflected in the scoring. Several different physiotherapists performed the M MAS-UAS −95 which could be a limitation, in spite of the fact that they were very familiar with the scale. On the other hand, all evaluations of arm and hand function were made as early as within 72 hours after stroke onset, which to our knowledge is not reported elsewhere.

This study is unique in assessing all patients with first ever stroke in a specific geographical catchment area, with an early examination of upper extremity function (within 72 hours after stroke onset) and exploration of the associations between impaired upper extremity function and outcome.

## Conclusion

As a conclusion; in a non-selected population of patients with first ever stroke, impaired arm and hand function is present in 48%, estimated within 72 hours after stroke onset. This is less than shown in previous studies and could indicate that the population of patients with stroke has changed. This study also shows that impaired upper extremity function was significantly associated with higher age, longer stay and higher mortality in the stroke unit care, as well as lower chance of discharge directly home.

## Competing interests

The authors declare that they have no competing interests.

## Authors’ contributions

HCP contributed to the study design, acquisition and analysis of data, drafting and competition of the manuscript. MP contributed to acquisition of data and revised the manuscript. AD contributed to the concept and study design, and revised the manuscript critically. KSS contributed the concept and overall study design, acquisition of data, manuscript editing. All authors’ have read and approved the final version submitted for publication.

## Pre-publication history

The pre-publication history for this paper can be accessed here:

http://www.biomedcentral.com/1471-2377/12/162/prepub
